# The Relationship between Executive Functions and Body Weight: Sex as a Moderating Variable

**DOI:** 10.3390/bs14030258

**Published:** 2024-03-21

**Authors:** Ciro Rosario Ilardi, Antonietta Monda, Alessandro Iavarone, Sergio Chieffi, Maria Casillo, Antonietta Messina, Ines Villano, Giovanni Federico, Vincenzo Alfano, Marco Salvatore, Walter Sapuppo, Vincenzo Monda, Marcellino Monda, Girolamo Di Maio, Marco La Marra

**Affiliations:** 1 IRCCS SDN—Istituto di Ricovero e Cura a Carattere Scientifico, SYNLAB Istituto di Diagnostica Nucleare, 80143 Napoli, Italy; 2 Department for the Promotion of Human Science and Quality of Life, Telematic University San Raffaele, 00166 Rome, Italy; 3 Neurological and Stroke Unit, Centro Traumatologico Ortopedico Hospital, AORN “Ospedali dei Colli”, 80131 Naples, Italy; 4 Department of Experimental Medicine, University of Campania “Luigi Vanvitelli”, 80138 Naples, Italy; 5 Department of Precision Medicine, University of Campania “Luigi Vanvitelli”, 80138 Naples, Italy; 6 Department of Wellness, Nutrition and Sport, Telematic University Pegaso, 80143 Naples, Italy; 7 Department of Psychology, Sigmund Freud University, 20143 Milan, Italy; w.sapuppo@milano-sfu.it; 8 Department of Economics, Law, Cybersecurity and Sports Sciences, University of Naples “Parthenope”, 80133 Naples, Italy

**Keywords:** obesity, executive functions, sex differences, Body Mass Index, waist circumference, moderation analysis

## Abstract

This study explores the interplay between executive functions and body weight, examining both the influence of biological factors, specifically sex, and methodological issues, such as the choice between Body Mass Index (BMI) and waist circumference (WC) as the primary anthropometric measure. A total of 386 participants (222 females, mean age = 45.98 years, SD = 17.70) were enrolled, from whom sociodemographic (sex, age, years of formal education) and anthropometric (BMI and WC) data were collected. Executive functions were evaluated using the Frontal Assessment Battery–15 (FAB15). The results showed the increased effectiveness of WC over BMI in examining the relationships between executive functions, sex differences, and body weight. In particular, this study revealed that there was a significant moderating effect of sex at comparable levels of executive functioning. Specifically, women with higher executive performance had lower WCs than their male counterparts, suggesting that executive function has a greater impact on WC in women than in men. Our findings highlight the importance of conducting more in-depth investigations of the complex relationship between cognitive deficits and weight gain, considering confounding variables of behavioral, psychobiological, and neurophysiological origin.

## 1. Introduction

Obesity affects about 650 million adults globally, transcending geographical boundaries. Given its contribution to the pathogenesis of several chronic diseases, within which it increases mortality rates [1,2,3,4,5], obesity represents a major public health challenge [6,7,8].

Over the past few decades, obesity has been associated with reduced cognitive performance. Consistently, it is now recognized as a risk factor involved in the onset of the majority of neurocognitive disorders [9,10,11,12,13,14,15,16,17,18,19]. 

Among the most extensively investigated cognitive domains, executive functions—higher-order cognitive functions associated with neural activity in the prefrontal cortex—appear to play a central role in the regulation of body weight. In general, these functions enable individuals to deal effectively with environmental challenges, particularly in novel or conflictual situations [20,21,22,23,24]. 

In the context of research on weight-related issues, previous behavioral studies have found that executive functions may predict body weight variability, dietary inhibition, appetite regulation, energy expenditure, adherence to a healthy diet, and outcomes of interventions aimed at weight reduction [25,26,27,28,29,30]. Congruently, neurophysiological evidence has identified the prefrontal cortex as a critical hub in regulating dietary self-control, operating in tandem with the mesocorticolimbic pathway to modulate reward mechanisms and the hedonic aspects of food choices [31,32,33]. Still, previous research in cognitive neuroscience has highlighted a significant association between impaired working memory and diminished gray matter volume in the left dorsal striatum, as well as alterations in the white matter microstructure of the left inferior longitudinal fasciculus along the occipitotemporal/ventral visual stream in individuals with higher body weight [34,35]. Given the involvement of the dorsal striatum in learning action–reward associations [36,37] and the role of the ventral visual stream in reward-cueing-related processes during spatial working memory tasks [38], this pattern of results may imply a significant relationship between reward processing mechanisms and obesity-related phenomena. 

Other studies have focused on decision making. For example, structural neuroimaging studies in obese individuals have reported reduced gray matter density in key frontal regions responsible for decision making, such as the frontal operculum, middle frontal gyrus, and orbitofrontal cortex [39,40,41]. In addition, a previous fMRI study on obese women participating in a monetary decision-making task showed that greater task complexity was associated with increased activation in the frontal cortex. Furthermore, reduced brain activity in the same regions predicted an increased rate of weight gain over the following 3 years [42]. Taken together, this brief overview only partially explains the relationship between executive functions, brain activity, and obesity.

Noteworthy are the “paradoxical” findings, such as those underpinning the so-called “obesity paradox”. This paradox implies a potential positive association between obesity and psychophysical well-being, including cognitive functioning, particularly in the older adult population [43,44,45]. Once again, these counterintuitive findings not only highlight the intrinsic complexity of the relationship between cognitive functions, obesity, and health status but also suggest the need for further research aimed at exploring this topic [46,47,48,49,50,51,52,53]. 

A number of factors can definitely influence the relationship between executive functions and obesity. These include the different methods used for assessing body weight and the role of some confounding variables [54,55]. The Body Mass Index (BMI), which is commonly used to assess weight status, has faced criticism as a more imperfect indicator for individual evaluation, particularly when measuring regional adiposity [56,57,58,59]. This may lead to distortions in evaluating obesity-related effects [58]. In contrast, waist circumference (WC) appears to be a more sensitive measure of visceral obesity, with greater reliability in predicting obesity-related health risk factors, such as cardiovascular diseases, diabetes, and mortality [60,61,62,63]. 

In addition to methodological issues, the interaction between executive functions and obesity may be further complicated by biological sex differences [64,65,66]. On the one hand, such differences may influence the prevalence of obesity; on the other hand, sex may influence raw performance in neuropsychological tests assessing executive functions [67,68,69,70,71,72]. Hence, it should be recommended to treat sex as a potential covariate to be kept under control in psychometric models attempting to explore the association between executive functions and obesity. 

The impact of sex on obesity prevalence is a stark global reality. Obesity is more prevalent among women in several geographic regions, including Sub-Saharan Africa, East Asia, the United States, and Europe [64,65,66]. This likely results from the interplay of sociocultural, environmental, and genetic factors [73]. Regarding neuropsychological outcomes, different studies have detected a significant concomitant effect of sex on tests exploring executive functioning. For instance, men and women exhibit distinct abilities in specific executive subdomains such as attention, planning, inhibition, and verbal fluency, and these differences manifest across different life stages [67,68,69,70,71,72]. In particular, increased impulsivity and shorter reaction times have been found in men as compared with women [68,72,74]. Conversely, better planning [75,76,77], attention [75,77], decision making [78,79], and working memory skills have been observed in female as compared with male individuals [80,81,82,83]. Nevertheless, it is important to stress that sex differences in executive functioning do not necessarily imply systematic disparities between sexes; rather, they may reflect differences in the cognitive strategies employed when required to perform a cognitive task [84,85].

Sex differences in neuropsychological test scores may extend to a lower level of abstraction and relate to variability in anatomofunctional brain characteristics. Indeed, men and women seem to employ different brain networks when performing a number of cognitive tasks, and may exhibit different activity across neurotransmitter systems, including dopamine and serotonin [86,87,88,89,90,91]. 

The present study was designed to further explore the intricate relationship between executive functions and body weight, taking into account the critical issues highlighted above. In particular, two primary objectives were outlined: (1) to assess the predictive value of executive functions in explaining the variance of different anthropometric measures (BMI vs. WC) and (2) to explore the potential moderating role of sex in the relationship between executive functions and body weight. In this regard, we expect that (1) executive functions will show a significant association with anthropometry and (2) sex will significantly moderate the relationship between executive functions and body weight. More specifically, we hypothesize that higher executive performance will be associated with lower body weight. Furthermore, we predict that sex will demonstrate a moderating effect on the association between executive functions and body weight.

## 2. Materials and Methods

### 2.1. Participants

A total of 386 participants (222 females) were involved in this study. Italian volunteers were recruited through the convenience sampling method from various districts in the Campania, Calabria, and Puglia regions of Southern Italy. The inclusion criteria for participation in this study were as follows: being at least 18 years old and having a minimum of 5 years of formal education (equivalent to completing primary school). Moreover, according to the normative datasets detailed in the Materials and Procedure section, participants who scored insufficiently on the cognitive batteries administered were excluded from the study. Additional exclusion criteria were past or current history of intellectual and/or linguistic deficits, or neurocognitive, psychiatric, or psychopathological disorders. None of the participants had a history of alcohol or substance abuse/addiction, and they were not receiving treatment with drugs known to affect cognitive performance. To prevent a “hyper normality” bias [92], individuals with chronic medical conditions that were managed pharmacologically, such as hypertension, cardiovascular diseases, or type II diabetes, were not excluded. 

### 2.2. Materials and Procedure

Participants were accommodated in a soundproof room where anamnestic information was collected, and anthropometric and neuropsychological measurements were performed. Specifically, the initial phase involved gathering sociodemographic data such as sex, age, and years of formal education. This was followed by anthropometric assessments, including measurements of weight, height (measured using a weight scale with a stadiometer), and waist circumference (WC). The Body Mass Index (BMI) value was calculated using Quetelet’s formula (expressed as kg/m^2^). WC was measured by encircling a tape measure horizontally around the abdomen, positioned approximately 2 cm above the navel. Global cognitive functioning was assessed by administering the Mini-Mental State Examination [93,94]. Executive functions were evaluated using the Frontal Assessment Battery–15 (FAB15) [69]. This is a short screening battery designed to assess general frontal/executive functioning. The FAB15 explores various executive domains, such as abstraction, generativity, set-shifting, planning, sensitivity to interference, and inhibitory control. The battery showed a robust psychometric architecture, supported by adequate internal consistency (Cronbach’s alpha = 0.72), a parsimonious unifactorial structure, and excellent interrater (ICC = 0.99) and test–retest (ICC = 0.98) reliability. Regression-based normative data are available from a cohort of 1187 healthy individuals [69]. The scoring range is 0–15, with higher scores indicating a better general executive functioning. Following correction of raw scores for sex, age, and education, participants scoring below 23.8 on the MMSE and 9.36 on the FAB15 were excluded. 

### 2.3. Statistical Analyses

Descriptive statistics were expressed as frequency for categorical variables and mean (M) and standard deviation (SD) for continuous variables. According to biological sex, between-group equivalence in age, education, and FAB15 scores was checked by univariate Analysis of Variance (ANOVA). Theoretically speaking, considering the potential differences in body composition between men and women, including the distribution of adipose tissue (specifically, visceral adiposity around the abdomen), sex disparities might be more pronounced in WC than BMI measurements. To mitigate this bias, we used a standardized regression-driven psychometric algorithm to remove the effects of possible linear associations between sex and anthropometric measures. Specifically, two simple linear regression models were constructed by independently entering BMI and WC as outcome variables and sex as a predictor. Based on the results of regression analyses, the following correction equation was solved:

$$antropometric\;adjusted\;value=antropometric\;value_{i}-\left[b_{sex}\times \left(x_{i\left(sex\right)}-{\bar{x}}_{\left(sex\right)}\right)\right]$$

where b refers to the unstandardized regression coefficient, which represents the average change in the dependent variable associated with moving from one sex category to the other, and x to the sex dummy variable (coded as 1 = female, 0 = male). Subsequently, the SPSS Macro PROCESS was employed for running moderation analysis with 5000 bootstrap samples. Two moderation models were tested. BMI- and WC-adjusted values entered each model as outcome variables, the FAB15 score as a predictor, and sex as a moderator, respectively. The nominal α level = 0.05 was adjusted according to Bonferroni’s correction for multiple comparisons, if appropriate. Overall, statistical analyses were performed by means of IBM SPSS Statistics v. 27.

## 3. Results

### 3.1. Preliminary Data Analysis: Normality Assumptions and Missing Data 

Univariate outliers, i.e., z-scores higher than three in absolute terms, were deleted. Thereafter, univariate normality was assessed according to skewness and kurtosis indexes. In particular, values ranging from −2 to +2 indicate the absence of appreciable deviations from normality. For diagnostics of multivariate outliers, Mahalanobis’ distance was used. No multivariate outliers were detected. Multivariate normality was assumed by Mardia’s kurtosis coefficient. 

### 3.2. Descriptive Statistics

Data from 386 participants (222 females; M age = 41.80 years, SD = 18.32, range = 18–86 years; M education = 15.11 years, SD = 3.96, range = 5–24) were analyzed. Mean BMI and WC values were 25.32 (SD = 4.53, range = 18.03–39.51) and 86.11 (SD = 14.83, range = 60–130), respectively. The mean MMSE score was 29.11 (SD = 1.29, range = 23–30) while the mean FAB15 score was 13.56 (SD = 1.62, range = 7–15). No sex differences were detected for age [F(1, 385) = 1.152, *p* = 0.284], education [F(1, 385) = 0.129, *p* = 0.720], MMSE [F(1, 385) = 0.110, *p* = 0.740], or FAB15 score [F(1, 385) = 0.909, *p* = 0.341]. As anticipated, no between-group differences were detected for BMI [F(1, 385) = 1.267, *p* = 0.21], while they emerged for WC [F(1, 385) = 20.910, *p* < 0.001]. Sex-group differences are reported in Table 1 for descriptive purposes. Based on the regression analyses’ results (BMI: $b_{\mathrm{sex}}$ = −0.525, SE = 0.47, t = −1.126, *p* = 0.26; WC: $b_{\mathrm{sex}}$ = −6.906, SE = 1.51, t = −4.573, *p* < 0.001), and taking into account an ${\bar{x}}_{\left(\mathrm{sex}\right)}$ of 0.58, correction equations were derived, par condicio, to adjust both BMI and WC values. In both instances, this led to a significant selective reduction in sex differences (*p*s > 0.99). 

### 3.3. Moderation Analysis

**Figure 1:**
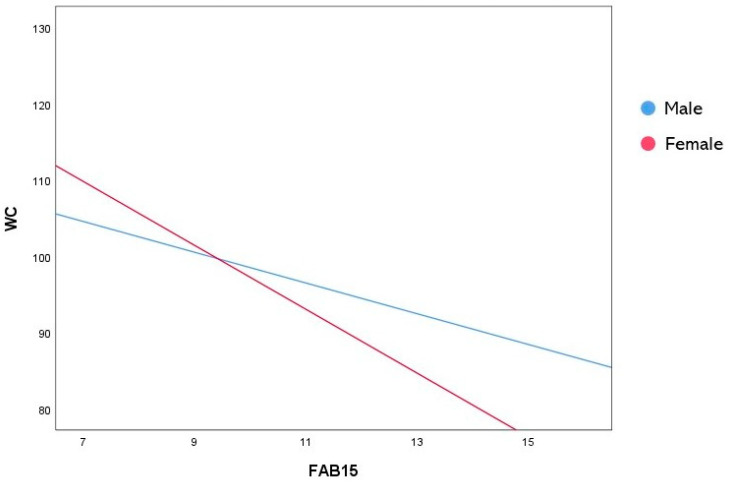
Interaction plot representing the significant moderating effect of sex in the relationship between FAB15 and WC. FAB15: Frontal Assessment Battery–15; WC: waist circumference.

To test the moderating role of sex in the relationship between the FAB15 and anthropometric measures, two moderation analyses with 5000 bootstrap samples were performed, where BMI- and WC-adjusted values were entered as dependent variables, respectively. The first model was statistically significant (R^2^ = 0.172, F(3, 382) = 22.802, *p* < 0.001) and showed a main effect of the FAB15 score only (b = −0.787, 95% CI [−1.246, −0.329], SE = 0.233, t = −3.376, *p* < 0.001) (Table 2). Similarly, the second model explained a significant amount of WC variance (R^2^ = 0.188, F(3, 382) = 25.324, *p* <0.001). In addition, both a main effect of the FAB15 score (b = −2.014, 95% CI [−3.426, −0.603], SE = 0.717, t = −2.808, *p* = 0.005) and a significant interaction between sex and the FAB15 (b = −2.172, 95% CI [−3.917, −0.427], SE = 0.887, t = −2.448, *p* = 0.015) were found (Table 3). This result suggests that sex significantly moderated the relationship between the FAB15 and WC. More specifically, as revealed by the analysis of the conditional effects at each moderator’s level (Table 4), female subjects with higher FAB15 scores had lower WCs than males (Figure 1). 

## 4. Discussion

In the current study, we examined the relationship between executive functioning, sex differences, and two different anthropometric indexes, namely BMI and WC. Our results showed the following: (1) executive functions, as measured by the FAB15, are significantly associated with body weight; (2) WC is a more reliable measure than BMI for examining the relationship between FAB15 score, sex, and body weight; and (3) biological sex exerts a moderating effect in the relationship between FAB15 score and WC. Specifically, we found that the association between waist circumference and the FAB15 was more significant in women, suggesting that executive function has a greater impact on WC in women than in men. Taken together, these findings provide additional insights into the interplay between executive functions and body weight. Considering the growing scientific interest regarding this topic [95,96,97,98,99], our results may offer meaningful contributions to this area of discussion by incorporating the role of sex-related physiological variability. However, only a few studies have explored this issue in the scientific literature [100]. Therefore, the interpretation of our findings draws predominantly from the realm of speculation and thus requires further investigations. In fact, the hypotheses that will follow are built upon spurious pathways.

Executive functions rely on the activation of various brain regions, particularly the prefrontal cortex, encompassing both the medial [101,102,103] and orbitofrontal regions [104,105,106,107]. Overall, the prefrontal cortex plays a critical role in executive processes like attention, action planning, and working memory. Still, the prefrontal cortex relies on a delicate balance between excitatory glutamatergic neurons [108,109] and inhibitory GABAergic neurons to function properly [101,109,110]. Similarly, neurotransmitters such as dopamine, endogenous opioids [102,111,112], and acetylcholine [113] have been associated with proficient executive functioning. To understand how sex differences might interact with cognition, it is essential to discuss the physiological variations occurring in neurotransmitter systems, particularly within the prefrontal cortex and striatum. 

Previous research in animal models has highlighted innate differences in serotonin and dopamine levels in the prefrontal cortex between sexes [114], as well as different behavioral responses following excitation of nicotinic receptors [115]. Additionally, sex differences have been observed in the expression of dopaminergic receptors into the striatum [116]. In humans, fluoxetine, a selective serotonin reuptake inhibitor, has been shown to enhance attention in women but not in men [117]. From the perspective of cognitive functioning, and moving from the molecular level to the “raw” gray matter, it is important to acknowledge how sex differences in structural brain development may determine sex differences in executive functions. Indeed, during the early stages of brain development, there is a proliferation of neurons and synapses, followed by a process of synaptic pruning, in which less active neural connections are eliminated [118]. 

Sex differences in brain development, in terms of both timing and complexity, are evident, with variations observed in both cortical maturation [119] and neuronal structural complexity [120]. These variations may influence different cognitive and behavioral patterns in each sex. Still, microglia and astrocytes, which play a key role in synaptic pruning, interact with some components of the immune system, known as the complement system, including specific proteins such as C1q and C3 [121,122]. Sex differences have been shown in these components, which appear, moreover, sensitive to environmental factors including stress [123,124] and alcohol exposure [125].

In the field of cognitive neuroscience, the impact of sex hormones on both brain connectivity and cognitive abilities is widely recognized, exhibiting significant variations depending on age and sex. Specifically, in women, sex hormones may affect brain connectivity, especially during the natural aging process and menopause. This seems to have a detrimental effect on cognitive performance [126,127]. In men, elevated testosterone levels were associated with reduced performance in specific executive tasks involving domains like error monitoring and set-shifting [128]. This pattern of results highlights how sex differences may exert a selective impact on cognitive functions [129]. 

Focusing on the link binding sex differences and executive functions to obesity, the role played by the Central Autonomic Network (CAN) is likely pivotal. This network encompasses brain regions such as the insula and ventromedial prefrontal cortex, interacting with the cingulate cortex and sensorimotor areas. Such interactions exert a significant impact on both autonomic nervous system activation and central brain processing. Thus, the CAN’s neural activity influences cognition, with a particular focus on executive functions [100,130].

Intrinsic activity within the CAN shows variations between male and female individuals due to their respective metabolic and hormonal differences [129]. These variations affect reward sensitivity, potentially influencing eating behavior. Moreover, they may be further exacerbated by limited awareness of internal bodily signals (interoceptive awareness) and difficulties in impulse control [53,131]. The dynamics of these mechanisms, in analogy with Damasio’s Somatic Marker Hypothesis [132], suggest that eating behavior and weight control abilities may vary between sexes according to different levels of CAN activation. This would imply a difference in the ability to effectively evaluate the long-term consequences of dietary choices, with the potential for dysfunctional eating behaviors. However, it is important to note that this hypothesis requires further in-depth investigation. Despite our study’s results showing that female sex might be considered a “protective” factor in the context of weight-related cognitive issues, executive functions impacted body weight in both sexes. In this vein, research in the field of physiology has unveiled a potential mechanistic pathway involving metabolic, immune, and neurological systems [133,134,135]. 

It is well known that obesity is strongly linked to metabolic dysregulation [136]. The relationship between cognitive dysfunctions and body weight may involve the alteration of brain glucose metabolism and mitochondrial function in obese individuals [137,138]. In addition, these individuals demonstrate a higher uptake of brain fatty acids compared to their lean counterparts [139]. In this context, the impact of sex as a biological modifier is relevant. Indeed, sex differences have been found in glycolysis and mitochondrial metabolism [140,141].

Still, adipose tissue, through the production of cytokines such as interleukin (IL1ß, IL6), interferon γ (IFNγ), tumor necrosis factor α (TNFα), and monocyte chemoattractant protein 1 (MCP1), contributes to the establishment of a chronic low-grade inflammation [142,143,144,145,146]. This persistent state of inflammation exerts its influence on the blood–brain barrier, resulting in endothelial dysfunction, neuroinflammation, and heightened oxidative stress, all of which can culminate in cognitive deficits [147,148]. Given that the blood–brain barrier interfaces with the central nervous system, it constitutes a key element in bridging the gap between peripheral inflammation and weight-related cognitive blunting, potentially extending to executive functions.

It should not be overlooked that differences in the association between executive functions and body weight between men and women may arise from the interaction of environmental, cultural, and social factors. For instance, in Western cultures, pervasive societal ideals of thinness for women and muscularity for men may influence individuals’ perceptions of body image [149,150,151,152,153], potentially impacting executive functioning differently between sexes. Conversely, in many African societies, where larger body sizes are often favored in women [154,155,156], different outcomes in executive functions may be observed in the female sex due to greater body satisfaction in response to greater body fat. 

Notwithstanding the aforementioned hypotheses, it is crucial to underscore that sex differences in the relationship between executive functions and body weight may be attributed to sex-related variability in strategies for task resolution and preferences for different outcomes rather than clear disparities in cognitive abilities between sexes [85]. In this regard, note that our assessment of executive functions exclusively relies on the FAB15. While this constitutes a neuropsychological battery, we cannot disregard the possibility that alternative measures, particularly domain-specific ones, may provide different insights, given the multifaceted nature of executive functions.

## 5. Conclusions

In this study, we found that WC may serve as a more effective body weight index than BMI in exploring the relationship between executive functions, sex, and body weight. Furthermore, our moderation model unveiled a significant modulation effect of sex. In particular, female participants, who scored higher in executive function assessments, exhibited a comparatively lower standardized WC than their male counterparts. This suggests that the relationship between executive function and WC is more pronounced in women than in men. These observations might be particularly relevant in the current scientific context, where the link between executive functions and obesity remains an area of active research and inquiry.

## Figures and Tables

**Table 1:** Mean (SD) by sex groups.

	Whole Sample (N = 386)	Females (*n* = 222)	Males (*n* = 164)	*p*-Value
Age (years)	41.80 (18.32)	42.66 (18.18)	40.63 (18.50)	ns
Education (years)	15.11 (3.96)	15.17 (4.03)	15.02 (3.89)	ns
MMSE	29.11 (1.29)	29.09 (1.40)	29.13 (1.12)	ns
FAB15	13.56 (1.62)	13.64 (1.72)	13.46 (1.47)	ns
BMI	25.32 (4.53)	25.10 (4.81)	25.62 (4.13)	ns
WC	86.11 (14.83)	83.26 (15.07)	90.17 (13.53)	<0.001

MMSE: Mini-Mental State Examination; FAB15: Frontal Assessment Battery–15; BMI: Body Mass Index; WC: waist circumference.

**Table 2:** Results of moderation analysis of BMI-adjusted-by-sex values.

	*b*	SE	t	*p*	LLCI	ULCI
Constant	36.213	3.159	11.462	<0.001	29.998	42.428
FAB15	−0.787	0.233	−3.376	<0.001	−1.246	−0.329
Sex	5.939	3.937	1.508	0.132	−1.806	13.685
FAB15×Sex	−0.476	0.289	−1.647	0.100	−1.045	0.092

FAB15: Frontal Assessment Battery–15; LLCI: Lower Level of Confidence Interval; ULCI: Upper Level of Confidence Interval.

**Table 3:** Results of moderation analysis of WC-adjusted-by-sex values.

	*b*	SE	t	*p*	LLCI	ULCI
Constant	118.653	9.702	12.229	<0.001	99.567	137.739
FAB15	−2.014	0.717	−2.808	0.005	−3.426	−0.603
Sex	20.444	12.065	1.694	0.091	−3.290	44.179
FAB15×Sex	−2.172	0.887	−2.448	0.015	−3.917	−0.427

FAB15: Frontal Assessment Battery–15; LLCI: Lower Level of Confidence Interval; ULCI: Upper Level of Confidence Interval.

**Table 4:** Results of analysis of conditional effects of FAB15 scores at sex levels.

	*b*	SE	t	*p*	LLCI	ULCI
Male	−2.014	0.717	−2.808	0.005	−3.426	−0.603
Female	−4.187	0.522	−8.023	<0.001	−5.213	−3.160

LLCI: Lower Level of Confidence Interval; ULCI: Upper Level of Confidence Interval.

## Data Availability

The data presented in this study are available on request from the corresponding author.
